# Anatomical investigation of safety determining factors for keyhole drilling trajectories for robotic cochlear implant surgery

**DOI:** 10.1007/s00405-024-09198-4

**Published:** 2025-01-17

**Authors:** Saliha Seda Adanır, Piraye Kervancıoğlu, İlhan Bahşi, Mohammad Al Saadi, Paul Van de Heyning, Vedat Topsakal

**Affiliations:** 1 Department of Anatomy, Faculty of Medicine, Gaziantep University, Gaziantep, Turkey; 2 Department of Otorhinolaryngology Head and Neck Surgery, University Hospital UZ Brussel, Vrije Universiteit Brussel, Brussels, Belgium; 3 Department of Otorhinolaryngology and Head and Neck Surgery, University Hospital Antwerpen, University of Antwerpen, Antwerp, Belgium; 4 Vrije Universiteit Brussel, Brussels Health Centre, Brussels, Belgium

**Keywords:** Cochlear implantation, Facial nerve anatomy, Facial recess, Robot-assisted cochlear implantation surgery, Sensorineural hearing loss, Surgical anatomy of temporal bone

## Abstract

**Purpose:**

Cochlear implants (CI) are the most successful bioprosthesis in medicine probably due to the tonotopic anatomy of the auditory pathway and of course the brain plasticity. Correct placement of the CI arrays, respecting the inner ear anatomy are therefore important. The ideal trajectory to insert a cochlear implant array is defined by an entrance through the round window membrane and continues as long as possible parallel to the basal turn of the cochlea. Image-guided surgery can directly drill with a robotic arm through the mastoid and execute an exact drilling of this precalculated most ideal trajectory. Here, we aim to identify critical anatomical structures determining the safest keyhole drilling trajectory by comparing easy and difficult CI surgeries performed with the HEARO Procedure: a robotic tool for image-guided surgery.

**Methods:**

Cone-beam computed tomography images of patients who underwent robot-assisted cochlear implantation surgery (RACIS) were included. Three of 25 cases had to be converted to conventional surgery because of the current safety mitigations based on anatomical distance. Radiological images in DICOM format were transferred to dedicated software (*OTOPLAN® Cascination GMHB Bern Switzerland*) for analyses. Surgical segmentation and previously planned trajectories were analyzed for these 25 cases by comparing cochlear sizes, facial recess sizes, round window sizes, and trajectory angles. In addition, facial recess angle, and cochlear orientation angles were measured with RadiAnt DICOM Viewer (*Medixant*,* Pozan*,* Poland*).

**Results:**

Facial recess size, facial recess angle, and distance between the facial nerve and safe trajectory were smaller in patient who converted from robotic surgery to conventional. A significant positive correlation existed between basal turn angle and in-plane angle *(p = 0.001, r = 0.859)*. In addition, there was a significant negative correlation between the basal turn length and the last electrode insertion depth degree (*p = 0.007, r = 0.545*).

**Conclusion:**

In our robotic surgery cases, we demonstrate that the limiting factors of anatomical relationships are constituted by the dimensions of the facial recess and the cochlear orientation. The findings of this study are considered to be a reference for future studies in achieving collision-free trajectory planning in robotic-assisted cochlear implant surgery.

## Introduction

Sensorineural hearing loss (SNHL) is not only a hearing deficit but also a severe social burden for affected patients, with sociocultural consequences for society as a whole [1]. The vast majority of SNHL cases are caused by inner ear dysfunction, which does not impact the larger anatomy of the cochlea. This allows the cochlea to remain tonotopically stimulable by cochlear implants [2]. Cochlear implants (CI) are the state-of-the-art therapy for patients with severe to profound SNHL, effectively compensating for the social burden of deafness [2, 3]. The tonotopic anatomy of the auditory pathways and the plasticity of the brain to adapt to another strategy for hearing has made a cochlear implant one of the most successful bioprothesis in medicine [4, 5]. Approximately 65,000 CI surgeries are performed worldwide annually, with nearly 1 million CI users globally [6, 7]. Cochlear implantation surgery is a microsurgical procedure in which certain tasks are on a submillimeter scale and require reliable visual-tactile feedback [6, 8]. The electrode array is placed within the scala tympani and, the excess electrode lead is left coiled in the surgically drilled mastoid cavity. The stimulator-receiver is surgically placed under the skin of the temporal region [9]. Although today’s electrodes are highly flexible, surgeons must insert them deeply into cochlear spaces with care. Rigid electrodes, however, risk damaging the cochlear walls [10]. Cochlear implantation surgery traumas including penetration of the spiral ligament, bony spiral lamina fractures, rupture of the basilar membrane, and array translocation into the scala media and scala vestibuli have been reported [11–14]. To avoid such traumas, it is crucial to determine the ideal and safe trajectory before surgery and to provide the patient with the appropriate trajectory angle [15, 16]. The orientation of the cochlea that has been reported to have considerable variation is an essential parameter for the calculation of the ideal trajectory [17]. The angle between the midsagittal axis and the long axis of the cochlea determines the orientation of the cochlea at the skull base [18]. Surgeons should pay attention to cochlear duct length (CDL) to have complete maximum cochlear coverage by implant arrays. So that safe surgical access to the cochlea, trauma to electrode insertion, and preservation of residual hearing are closely related to cochlear orientation [19–21].

The mastoidectomy and posterior tympanotomy approach (MPTA) is considered the gold standard approach for cochlear implantation surgery [15]. Although it still has a very small risk of facial nerve (FN) injury (< 1%), it is a well standardized intervention needs different reference on facial nerve risk [5, 22]. Today the use of a commercially available facial nerve monitors is considered standard of care since 1985 [23].

Entering the inner ear via the round window (RW) is the most structure preserving (and probably residual hear preserving) technique. Over the years the golden standard for surgical approach has become the posterior tympanotomy combined with an atticoantor mastoidectomy allowing access through the facial recess (FR). For a MPTA a high-speed drill is used to gain access through the space called as FR between the facial nerve and the chorda tympani [8, 24]. It is essential to know FR sizes and the angle between the chorda tympani and facial nerve (FRA) beforehand to avoid facial nerve and chordae tympani injuries during the cochlear implantation surgery. The robot and navigation-assisted keyhole surgery defined by the HEARO procedure have by protocol risk mitigation not to drill closer than 0.4 mm to the facial nerve, whereas surgeons with conventional surgery can safely expose the facial nerve from its bony canal [5]. Once the tympanic cavity has been accessed, access to the cochlea occurs via the round window or cochleostomy to insert the electrode array. The size and shape of the RW reported in the literature is quite variable and most often not even round [25, 26]. If the RW anatomy is appropriate, insertion from the RW is thought to be the least traumatic approach [27]. The RW is covered by a membrane called the secondary tympanic membrane [28]. During access to the cochlea via the RW, this membrane must be punctured [27, 29]. Canonus is defined as a bony prominence that partially covers and protects the round window [29, 30]. In robotic surgery, this structure is drilled open as large as necessary to access with arrays towards round window (*canonostomy*). In conventional surgery, the canonus is completely removed by performing a *canonectomy* to allow the surgeon to insert and also estimate the angle of correct insertion, whereas in robot-assisted cochlear implantation surgery (RACIS) this is pre calculated and robot arm just executes. Therefore, it is essential to estimate the canonus thickness before the operation [15, 16, 29, 30]. Another important parameter for cochlear implantation surgery is the variable anatomy of the cochlea, which is of key importance for implantation [21]. It was reported that there is a significant relationship between the postoperative electrode insertion depth degree and cochlear morphometry [31]. Therefore, cochlear morphometry directly affects proper electrode selection and postoperative audiological outcomes.

If cochlear implant arrays become smaller than they are today they will be very difficult to handle for surgeons without robotic support. Because robots outperform human dexterity, robotic surgery for cochlear implantation is gaining popularity [32]. Robotic keyhole drilling towards inner ear requires stringent accuracy of robot arm and navigation system. Safe robotic systems also have customized and highly sensitive facial nerve monitoring systems [33].

Thorough knowledge of anatomy is of utmost clinical importance for surgeons. Especially in the case of robotic keyhole surgery where surgeons do not have any visual feedback and cannot go from one anatomical landmark to another the importance of image evaluation becomes clear. Image-guided and robot arm-assisted cochlear implant surgery may seem a very logical strategy to improve the outcomes of cochlear implant surgery. However, they not only impose a close collaboration between surgeons and engineers but also radiologists and anatomists to determine safe and optimal trajectory definitions in the pioneered stage these days. Automated segmentation of tomographs and probably artificial intelligence can be assistive in the future but today we need to delineate the definitions of safe trajectories in variable temporal bone anatomy.

Facial recess sizes and angle, canonus thickness, round window sizes and cochlear orientation in cases that were planned for robotic surgery are essential. We compared these anatomical structures between successful robotic surgeries and the converted cases to conventional surgery to delineate difficulties in anatomical relationships.

## Materials and methods

For the study, approval was obtained from Medical Ethics Committee UZ Brussel Hospital with number 1432022000187. VT holds a national FWO FKM senior researcher grant number 18B2222N.

### Patients

Preoperative and postoperative CBCT images of patients who underwent robot-assisted cochlear implantation surgery. All cases requiring cochlear implant surgery in an academic comprehensive cochlear implant center were screened for eligibility for RACIS. Intraoperative tomographs were analyzed and compared between successful RACIS cases and aborted cases. Anatomically challenging cases were excluded from these series such as incomplete partition of the cochlea. All other etiologies were included including some cases with intracochlear ossification) All patients were inserted with the same flexible electrode of 28 mm length (Flex 28, MEDEL Innsbruck Austria). The exclusion criterion was determined as images with low image quality in which anatomical landmarks determined for measurement could not be determined. Also, unsafe trajectories with a smaller facial recess than 2.5 mm were excluded.

### Robot-assisted cochlear implantation surgery (RACIS)

The HEARO procedure (CASCINATION Bern Switzerland etc.) an assistive otological set of tools was described elsewhere by was described by Topsakal et al.‘s [5]. While 3 of 25 patients were converted to conventional surgery as a result of intraoperative evaluation (*facial nerve monitoring*), 22 patients were completed with the HEARO procedure [5]. Intraoperative imaging could not guarantee a safe distance to the facial nerve in one of the 3 patients (*Subject 19*), the other 2 patients have been converted to conventional surgery because of software issue (In two cases, the intraoperative accuracy of the drilling trajectory could not be confirmed by OTOPLAN due to metal artifact or insufficient image contrast resolution and the procedure was converted to conventional) [5].

### 3D reconstruction of the cone beam tomographs of temporal bone

**Fig. 1:**
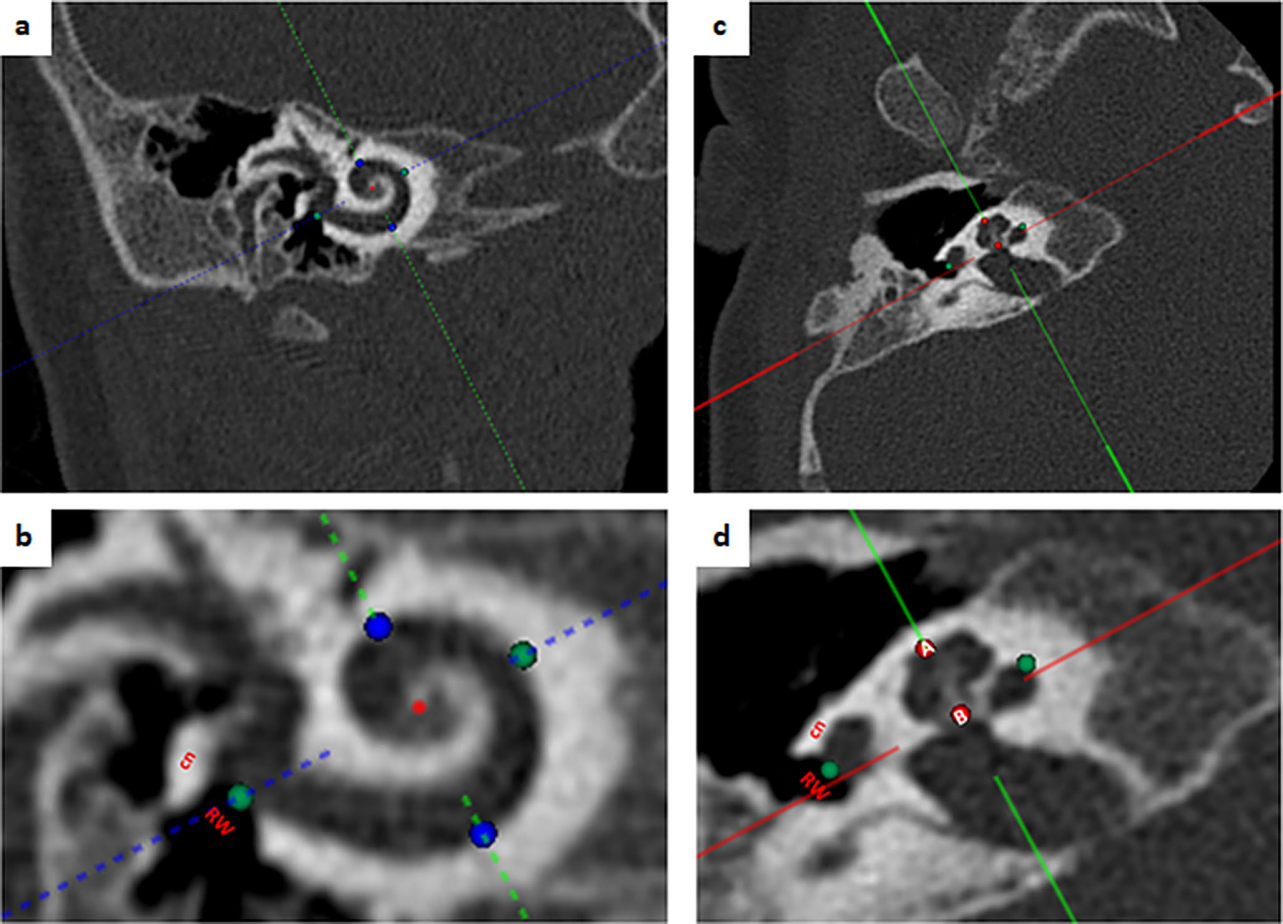
Measurement of the cochlear sizes. **a**) measurement of the cochlear length and width on the coronal view. **b**) landmarks for the measurement of the cochlear length and width. RW: round window, cn: canonus. **c**) measurement of the cochlear height on the axial view. **d**) landmarks for the measurement of the cochlear height. A: apex of the cochlea, B: base of the cochlea

OTOPLAN, (CASCINATION AG, Bern, Switzerland) was used for 3D reconstruction of the temporal bone, auditory ossicles, facial nerve, chordae tympani, cochlea and round window (Fig. 1). CBCT images in DICOM format were transferred to OTOPLAN. For the 3D reconstruction, anatomical landmarks have been determined in CT images and the following parameters have been measured.

### Measurements of cochlear sizes

Cochlear measurements have been performed in cochlear view. Cochlear view is the view defined as a double-oblique coronal view of the cochlea, in which the RW center is set to 0° with the coordinate system of the cochlea.

- ***Anatomical landmarks for cochlear length (CL)***: Distance between the centre of the round window and the furthest point on the opposite wall of the cochlea on coronal section of cochlear view (Fig. 1a-b).
- ***Anatomical landmarks for cochlear width (CW)***: Distance between inferior and superior point of the lateral wall on coronal section of cochlear view (Fig. 1).
- ***Anatomical landmarks for cochlear height (CH)***: Distance between the centre of the cochlear base and apex of the cochlea on the axial section of cochlear view (Fig. 1).
- ***Cochlear duct length (CDL)*** was measured by OTOPLAN automatically according to cochlear sizes with the method described by [34].
- ***Basal turn length (BTL)*** was calculated using a method proposed by Escudé et al. [35]. L = 2.62 × CL × loge (1.0 + θ/235) = 2.43 × CL.

### Measurements of round window sizes

Measurements have been performed in the coronal and axial sections of cochlear view.

**Fig. 2:**
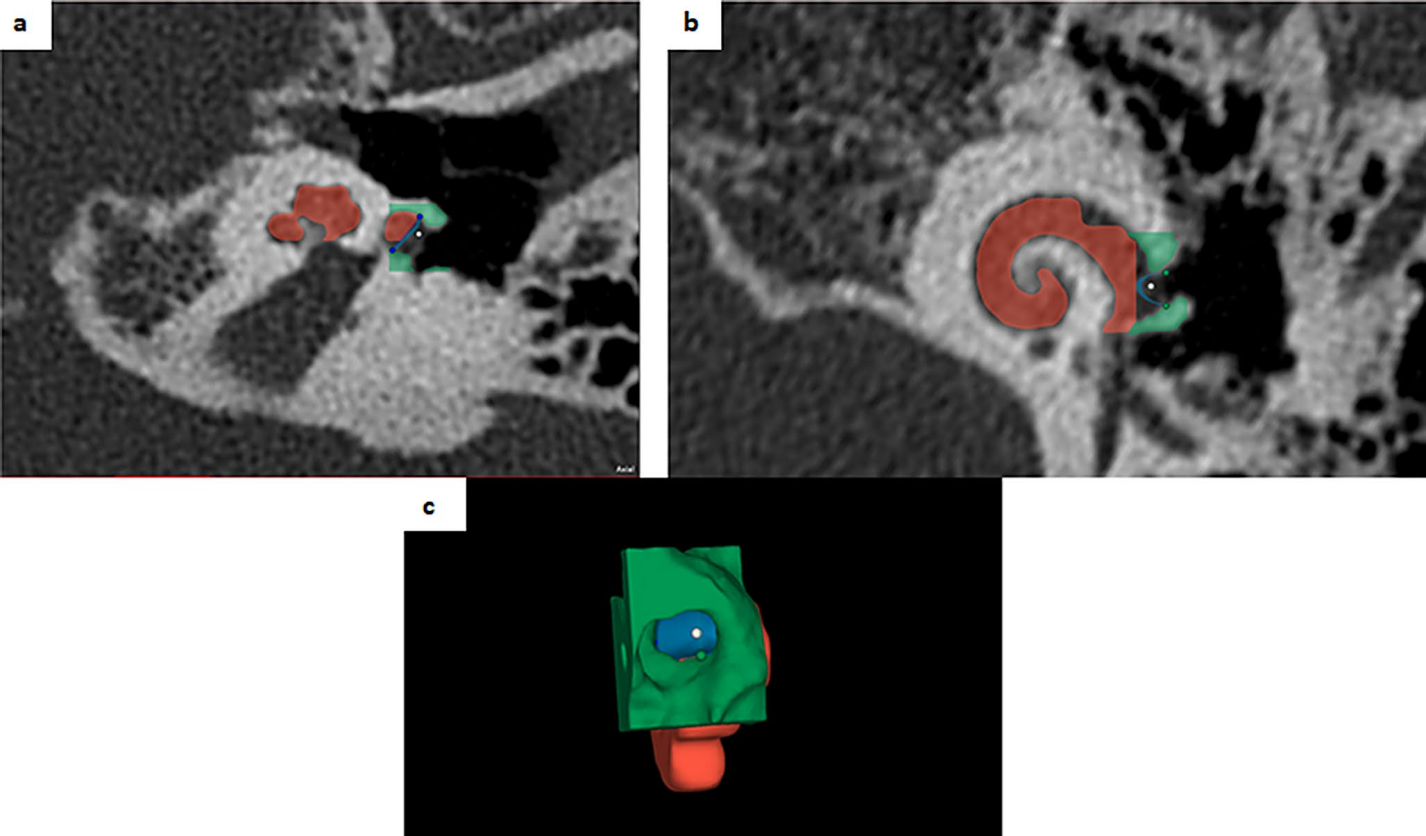
Measurement of the round window sizes. **a**) measurement of the supero-inferior diameter of the round window on the axial view. **b**) measurement of the antero-posterior diameter of the round window on the coronal view. **c**) 3D reconstruction of the round window

- ***Superior-inferior diameter of round window (RWSI)***: Distance between two end points (superior and inferior points) of round window on coronal view (Fig. 2).

- ***Anterior-posterior diameter of round window (RWAP)***: Distance between two end points (anterior and posterior points) of round window on axial view (Fig. 2).

### Measurement of canonus sizes

**Length of canonus (LC)**,** thickness of canonus (TC)** was measured by RadiAnt DICOM Viewer 2023.1.1 (*Medixant*,* Pozan*,* Poland*). For examiner reliability, measurements were made a second time by the same observer, one month apart.

**Fig. 3:**
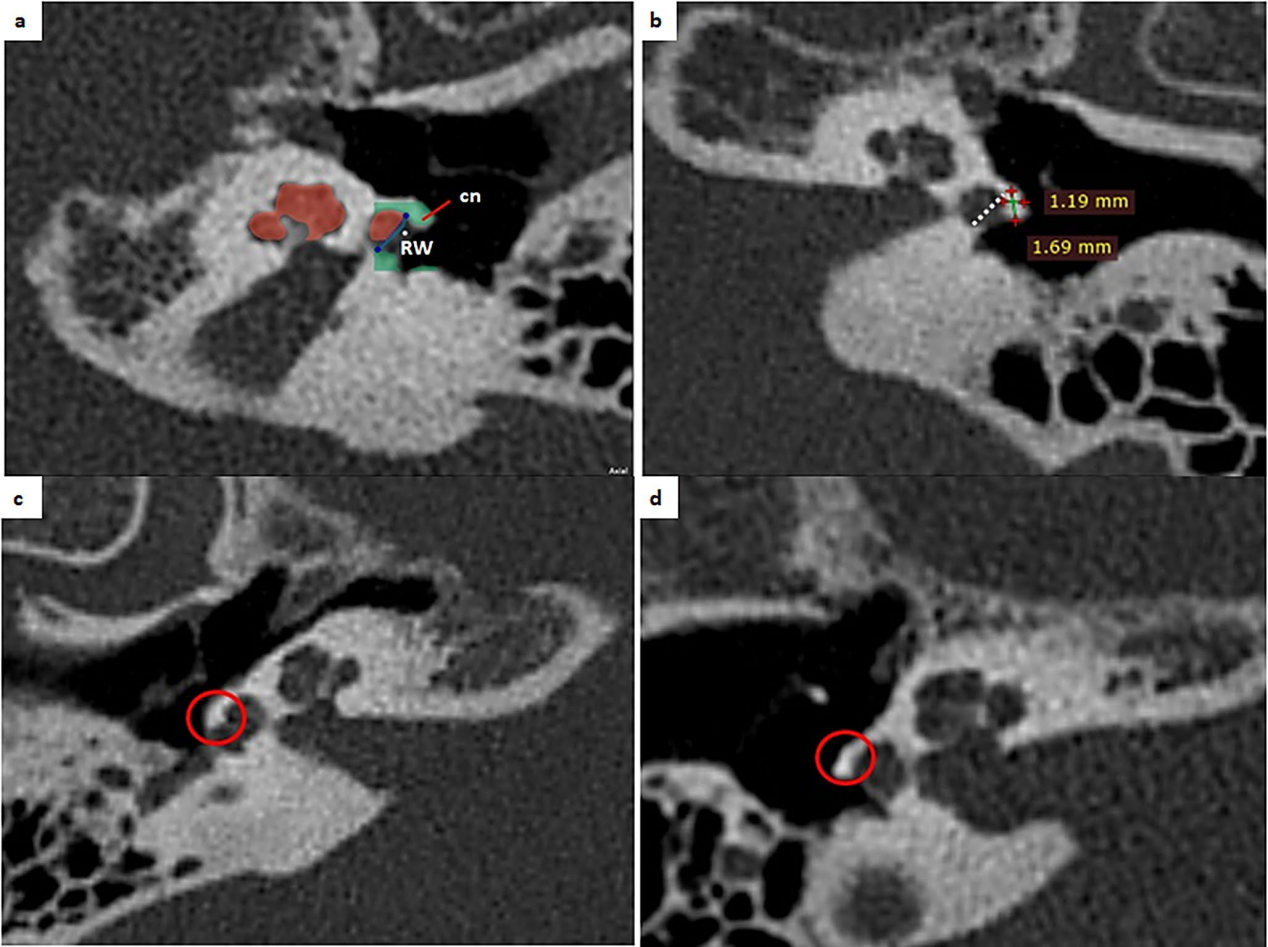
Measurement of the canonus sizes and typing of canonus. **a**) axial view of the cochlea, cn: canonus, RW: Round window, **b**) Measurement of the thickness of the canonus and length of the canonus, **c**) hook type canonus **d**) flat type canonus

- ***Length of canonus (LC)***: Distance between start and end point of canonus in axial section (Fig. 3b).

- ***Thickness of canonus (TC)***: Anteroposterior distance of the thickest part of canonus in axial section (Fig. 3b).
- ***Canonus type***: Canonus was typed as hook (Fig. 3c) or flat (Fig. 3d) according to its shape.

### Measurements of angles relations with cochlear orientation

Basal turn angle, cochlea apical angle was measured by RadiAnt DICOM Viewer 2023.1.1 (*Medixant*,* Pozan*,* Poland*) For examiner reliability, measurements were made a second time by the same observer, one month apart. (*OTOPLAN is a tool calibrated only for otological measurement and does not allow free measurement. For this reason*,* RadiAnt DICOM Viewer was used for angle measurements.)*

**Fig. 4:**
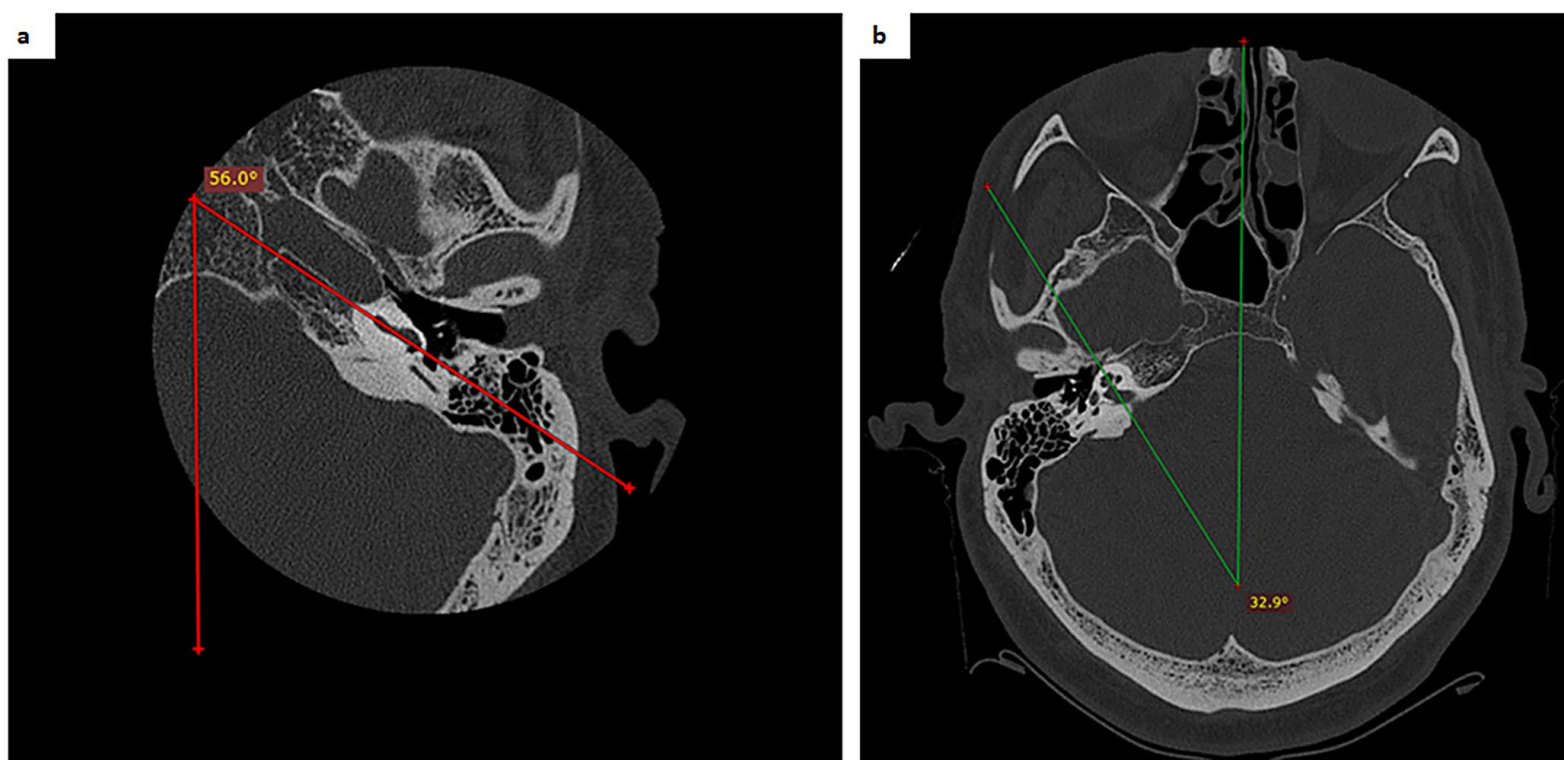
Measurements of the basal turn angle and cochlea apical angle on the axial view. **a**) basal turn angle, **b**) cochlea apical angle

- ***Basal turn angle (BTA)***: Angle between the line passing through the basal turn and the midsagittal plane (Fig. 4a).

- ***Cochlea apical angle (CAA)***: Angle between the line passing through the apex and the midsagittal plane (Fig. 4b).

### Measurements of facial recess width (FRW), facial recess angle (FRA) and depth of the facial nerve mastoid segment (DFNM)

**Fig. 5:**
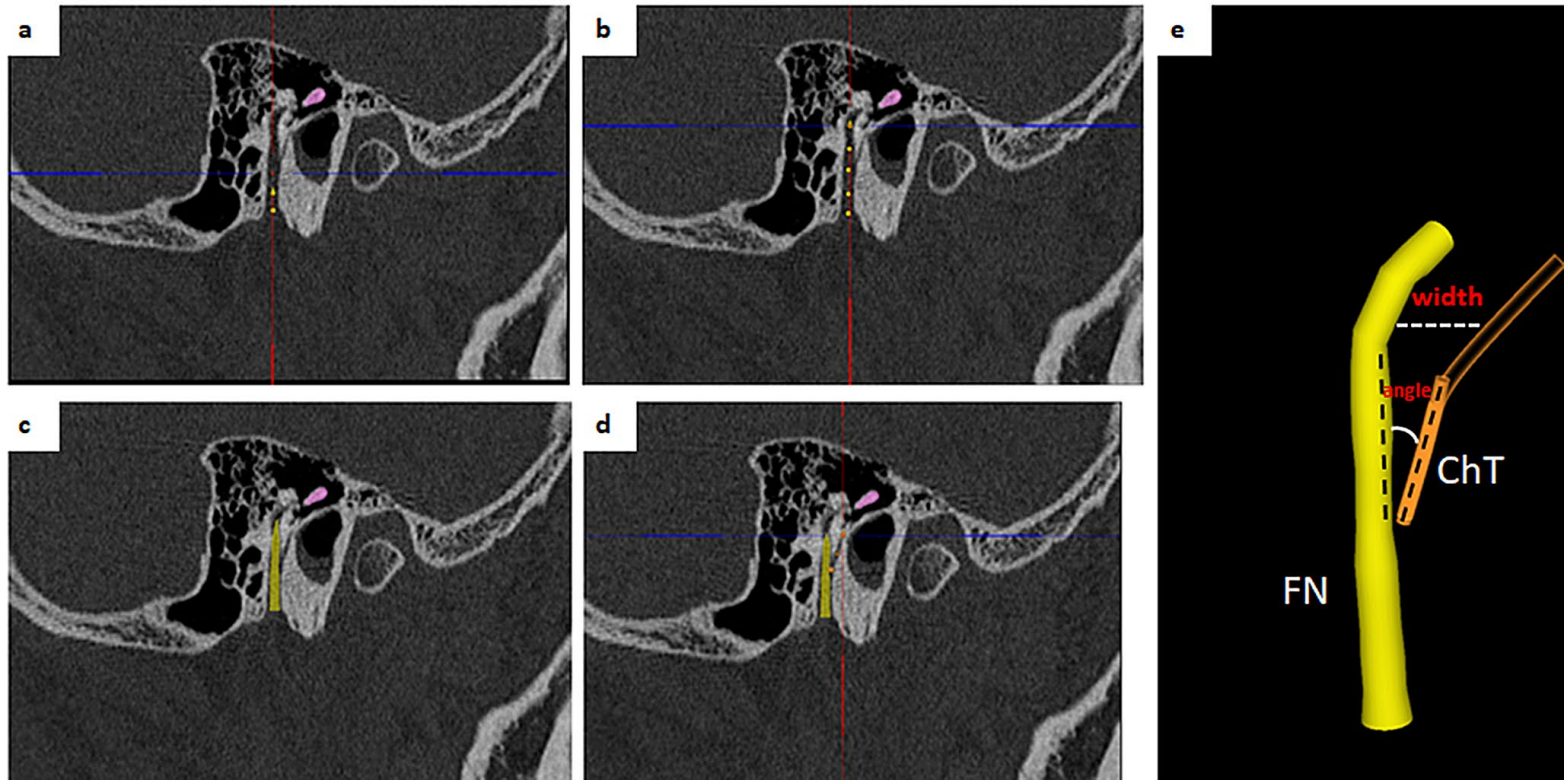
3D reconstruction of the facial nerve and chorda tympani on the coronal view. **a-b-c**) marking of the facial nerve along the facial canal **d**) marking of the chorda tympani from where it origins from the facial nerve **e**) 3D reconstruction of the facial recess

**Fig. 6:**
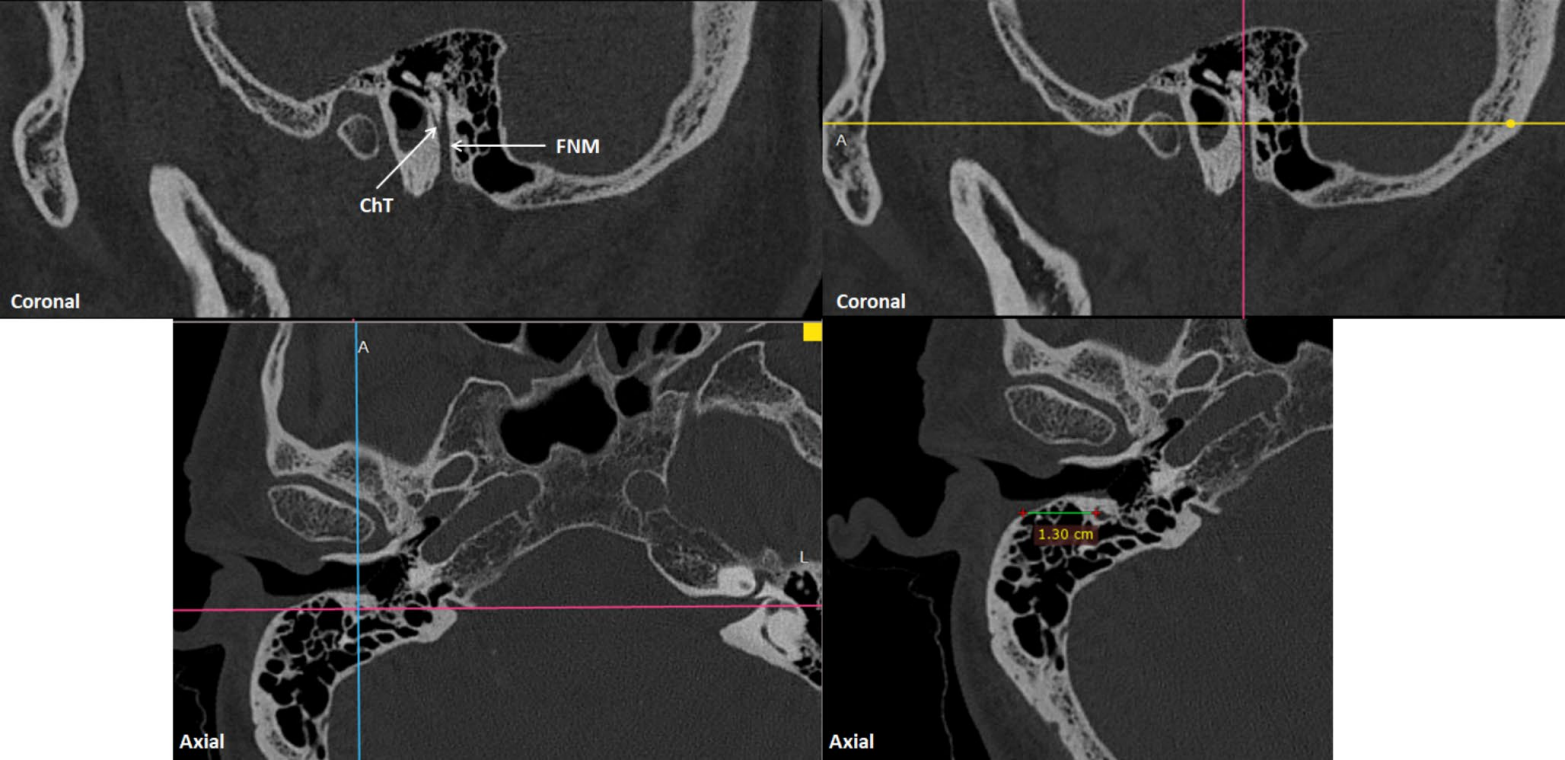
Measurements of the depth of the facial nerve mastoid segment

Facial recess width (FRW) was measured automatically with 3D reconstruction made by OTOPLAN (Fig. 5). Facial recess angle (FRA) was measured using a digital image analysis program (ImageJ; US National Institutes of Health, Bethesda, MD) on the 3D reconstruction image made via OTOPLAN. For angle measurement, in the 3D reconstruction image, the head was brought to the position where the trajectory would pass through the facial recess. The depth of the facial nerve mastoid segment (DFNM) has been measured on axial CT slices (Fig. 6). The coronal section was detected in which the mastoid segment of the facial nerve and the chorda tympani began to appear together for the first time. In the axial section corresponding to this section, the distance between the facial nerve mastoid segment and the lateral surface of the mastoid process has been measured. The measurement was made via RadiAnt DICOM Viewer 2023.1. For examiner reliability, measurements were made a second time by the same observer, one month apart.

### Calculation of the trajectory angles

**Fig. 7:**
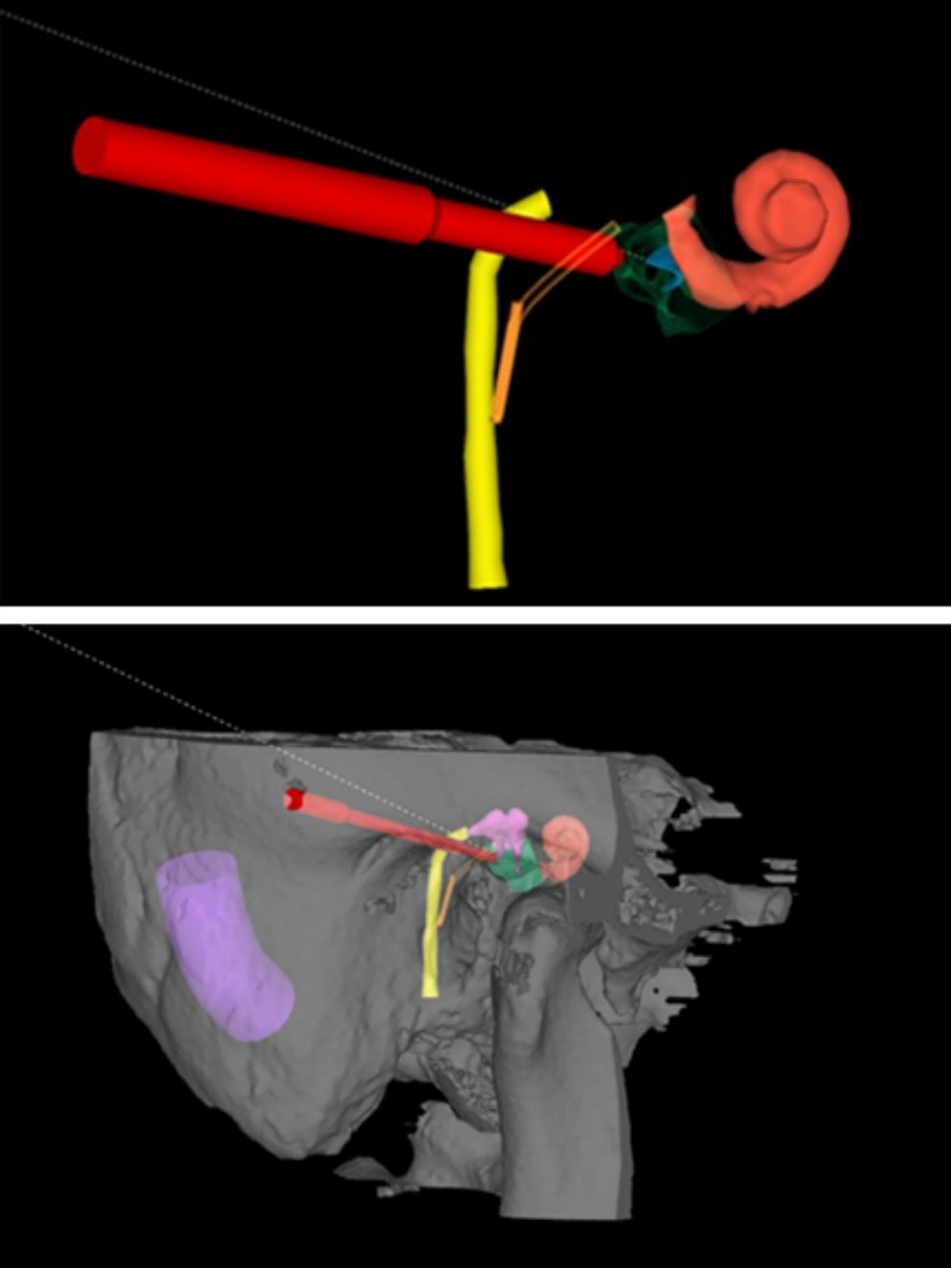
Calculation of the planned trajectory and in-out plane angles after the reconstruction of all structures

The ideal insertion trajectory aligns with the centerline of the scala tympani at the level of the round window membrane to avoid damage to the basilar membrane, modiolus, or spiral ligament during insertion [15]. Wimmer et al. [16] described two angles describing deviations from this ideal trajectory in two different planes. The in-plane can be defined as the angle between the planned trajectory and the axis passing parallel to the scala tympani in the inferior view. Consistently, the out-of-plane angle can be defined as the angle between the planned trajectory and the axis passing parallel to the scala tympani in the anterior view. The trajectory angles were calculated with OTOPLAN (Fig. 7).

### Calculation of the electrode insertion depth degree

**Fig. 8:**
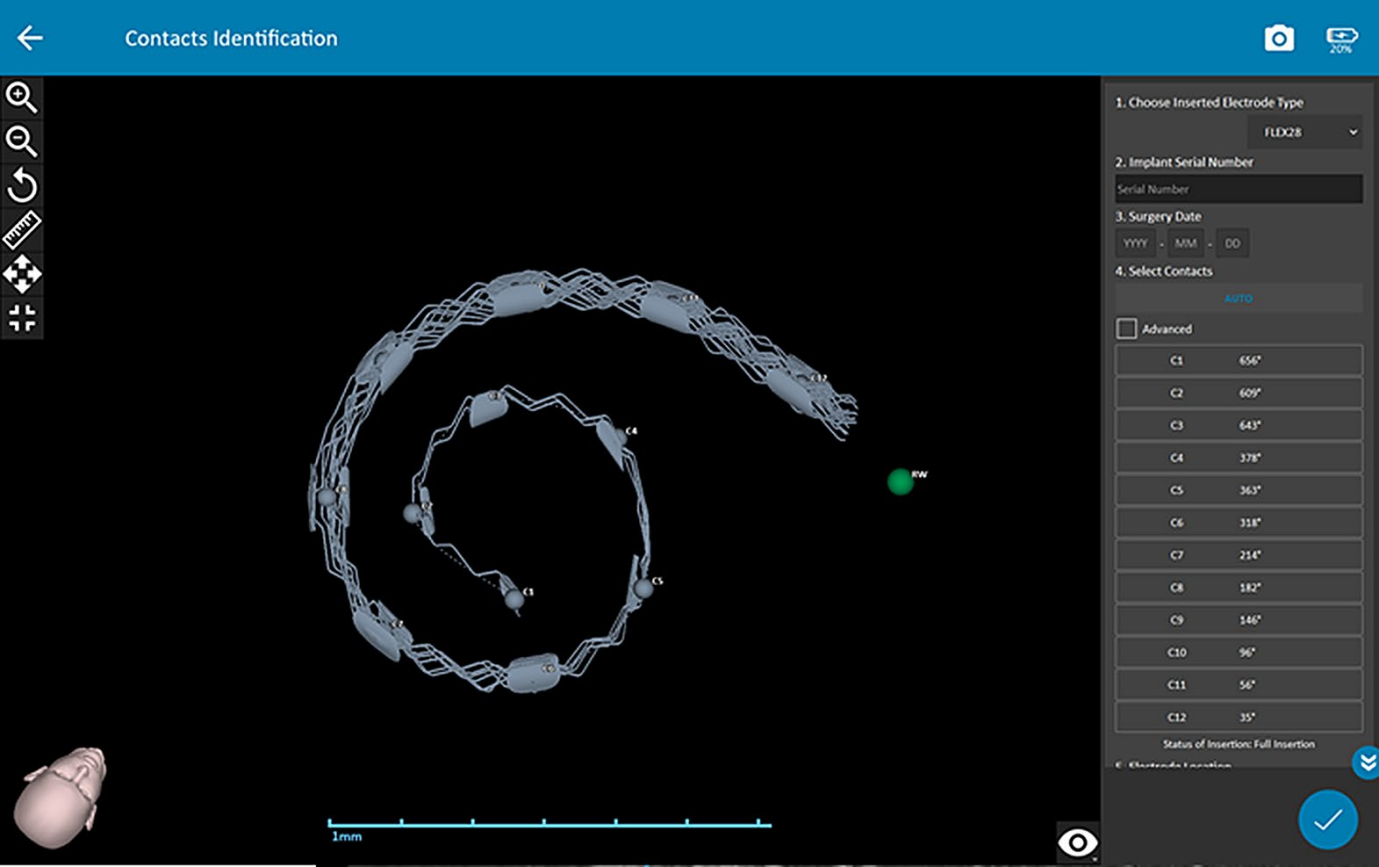
Calculation of the electrode insertion depth degree via the OTOPLAN

Electrode insertion depth degrees were calculated via OTOPLAN on postoperative CT images (Fig. 8).

### Statistical methods

Compliance of numerical data with normal distribution was tested with the Shapiro Wilk test. Mann-Whitney U test was used to compare variables without normal distribution in two groups. Relationships between numerical variables were tested with the Pearson correlation coefficient and the relationships between categorical variables were tested with the chi-square test. SPSS 22.0 package program was used in the analysis (IBM Corporation; Armonk, NY, USA). *P* < 0.05 was considered significant.

## Results

CBCT images of 25 (M:19, F:6, mean age: 56.72 ± 14.60) patients had robot-assisted cochlear implantation surgery were included in the study. The anatomical measurements of the patients are given in Table 1. Descriptive statistics of one patient (*Subject 19*) whose safe distance to the facial nerve could not be guaranteed with intraoperative imaging and 22 patients who completed robotic surgery have been given in the Table 2. Although the comparison between one case (subject 19) and 22 cases was not decisive, CAA, FRW, and FRA were smaller in subject 19 than in other cases.

**Table 1:** Anatomic measurements of the patients

Parameters		Total (*n*: 25)	Male (*n*: 19)	Female (*n*: 6)	*P*
		Mean ± SD	Mean ± SD	Mean ± SD	
Cochlear length (CL) (mm)		9.29 ± 0.43	9.37 ± 0.42	9.09 ± 0.43	0.357
Cochlear width (CW) (mm)		6.74 ± 0.48	6.84 ± 0.38	6.48 ± 0.63	0.130
Cochlear height (CH) (mm)		3.94 ± 0.38	3.98 ± 0.36	3.84 ± 0.46	0.354
Cochlear duct length (CDL) (mm)		35.83 ± 2.14	36.31 ± 1.65	34.59 ± 2.86	0.097
Basal turn length (BTL) (mm)		22.76 ± 1.01	22.78 ± 1.00	22.71 ± 1.11	0.929
Basal turn angle (BTA) (°)		58.72 ± 8.49	58.98 ± 9.49	58.05 ± 5.70	0.923
Cochlea apical angle (CAA) (°)		33.04 ± 7.02	33.61 ± 7.99	31.60 ± 3.57	0.423
Depth of facial nerve (DFNM) (mm)		17.58 ± 3.76	17.84 ± 3.22	17.21 ± 3.03	0.547
Facial recess size (FRS) (mm)		3.01 ± 0.48	3.15 ± 0.51	2.98 ± 0.33	0.235
Facial recess angle (FRA) (°)		34.04 ± 5.88	34.11 ± 6.12	33.86 ± 5.66	0.976
Length of canonous (LC) (mm)		3.41 ± 0.61	3.52 ± 0.65	3.11 ± 0.39	0.158
Thickness of canonous (TC) (mm)		1.29 ± 0.41	1.24 ± 0.40	1.43 ± 0.43	0.220
Canonous type	Flat	19	13	6	0.069
	Hook	6	6	0	
Superior-inferior diameter of round window (RWSI) (mm)		1.88 ± 0.37	1.95 ± 0.31	1.71 ± 0.49	0.198
Anterior-posterior diameter of round window (RWAP) (mm)		1.60 ± 0.49	1.70 ± 0.48	1.36 ± 0.45	0.244

SD: Standard deviation

**Table 2:** Comparison between subject 19 whose safe distance to the facial nerve could not be guaranteed with intraoperative imaging and 22 patients who completed robotic surgery

Parameters	Subject 19	RACIS (*n*: 22)
CL (mm)	9.86	9.26 ± 0.43
CW (mm)	7.05	6.71 ± 0.48
CH (mm)	3.93	3.94 ± 0.41
CDL (mm)	37.78	35.68 ± 2.12
BTL (mm)	23.96	22.68 ± 0.99
BTA (°)	64.40	59.31 ± 8.50
CAA (°)	26.17	32.36 ± 5.78
FRW (mm)	2.68	3.12 ± 0.32
FRA (°)	29.31	39 ± 6.55
DFNM (mm)	20.05	17.45 ± 3.82
LC (mm)	3.36	3.42 ± 0.63
TC (mm)	1.16	1.31 ± 0.42
RWSI (mm)	2.05	1.87 ± 0.39
RWAP (mm)	1.79	1.55 ± 0.50

BTL: Basal turn length, BTA: Basal turn angle, CL: Cochlear length, CW: Cochlear width, CH: Cochlear height, CDL: Cochlear duct length, CAA: Cochlea apical angle, DFNM: Depth of facial nerve, FRW: Facial recess width, FRA: Facial recess angle, LC: Length of canonous, TC: Thickness of canonous, RACIS: Robot-assisted cochlear implantation surgery, RWAP: Anterior-posterior diameter of round window, RWSI: Superior-inferior diameter of round window

### Trajectory analysis

The distance of the trajectory determined by 3D reconstruction to important anatomical points was calculated (Table 3). In Subject 19, the calculated trajectory distance to the facial nerve was smaller.

**Table 3:** Safety margin distance

Groups	Subject 19	RACIS (*n*: 22)
Stapes (mm)	1.01 ± 0.22	1.08 ± 0.45
Incus & Malleus (mm)	2.64 ± 0.43	2.72 ± 0.58
External auditory canal (mm)	1.57 ± 0.61	1.53 ± 0.65
Facial nerve (mm)	0.40 ± 0.12	0.51 ± 0.28
Chordae tympani (mm)	0.40 ± 0.20	0.43 ± 0.07

RACIS: Robotic assisted cochlear implantation surgery

### Trajectory angles

The mean in-plane angle was 6.54°±5.13°, and the out-plane angle was 19.01°±6.65°.

### Correlation between the trajectory angles and cochlear orientation

While a high degree significant positive correlation was found between basal turn angle and in plane angle (*p* = 0.001, *r* = 0.859), there was no significant correlation between other parameters (*p* > 0.05).

### Comparison between the 1 patient who had no full electrode insertion and the other patients

When the anatomical measurements of subject 17 who could not achieve full electrode insertion and other patients were compared, it was found that the superior-inferior diameter of round window (1 mm) of the subject 17 was smaller than the other patients (1.92 ± 0.33 mm).

### Postoperative electrode insertion depth degree

The electrode insertion depth degree of 3 RACIS patients with cochlear ossification and anomalies (*Cogan syndrome*,* IP III*,* post-meningitis ossification*) was not included in the mean electrode insertion analysis. The postoperative electrode insertion depth degree of one of the RACIS patients could not be calculated because the postoperative CBCT images of one patient was suitable. Additionally, full electrode insertion was achieved in all patients except one RACIS patient. The mean electrode insertion depth degree of the 18 patients is given in the Table 4.

**Table 4:** Postoperative electrode insertion depth degree

Electrodes	Insertion depth degree (°)
	(Mean ± SD)
C1	571 ± 65°
C2	500 ± 72°
C3	424 ± 66°
C4	355 ± 50°
C5	305 ± 38°
C6	261 ± 33°
C7	219 ± 32°
C8	177 ± 30°
C9	135 ± 28°
C10	95 ± 27°
C11	60 ± 24°
C12	29 ± 16°

### Correlation between the anatomical measurements and electrode insertion depth degrees

When the relationship between cochlear sizes and electrode insertion depth degrees was examined, a moderate significant negative correlation was found between the basal turn length (BTL) and the last electrode (e12) insertion depth degree (*p* = 0.007, *r*=-0.545). Other sizes (CL, CW, CH, CDL) had no statistically significant effect on electrode insertion depth in degrees (*p* > 0.05). There was no statistically significant relationship between BTA and CAA and postoperative electrode insertion depth degrees (*p* > 0.05).

## Discussion

Advancements in robotic interventions in otology require a thorough analysis of temporal bone anatomy, particularly in cochlear implantation, where success heavily depends on cochlear tonotopy. Surgical precision alone is insufficient without a comprehensive understanding of the underlying anatomy. Image-guided surgery is rapidly advancing the application of anatomical knowledge in clinical settings, particularly in cochlear implantation. However, many aspects of temporal bone anatomy remain unexplored, and the significance of anatomical structures increases with the introduction of technologies that necessitate detailed anatomical studies. In this study, we analyzed the temporal bone anatomy of patients with severe to profound deafness requiring cochlear implants. Our findings highlight that, even with the most accurate navigation systems and robotic arms available in healthcare, the complexity or ease of the surgery is ultimately dictated by the patient’s unique anatomical features.

The importance of atraumatic insertion is clear to every surgeon. But the ideal insertion trajectory and also speed remains to be discovered. Currently, the ideal trajectory is defined to go through the RW membrane and remains parallel to the basal turn as long as possible to avoid damage to lateral wall or modiolus in the basal turn. Any damage can induce molecular mechanisms that result in ossification which would give more resistance to the cochlear implant interface and eventually poorer functioning. Osteoprotegerin, for instance, is a protein with inhibitory regulatory functions. In Streptococcus pneumoniae infections, this regulatory cascade is disrupted, reducing inhibition and leading to cochlear ossification [36]. Additionally, surgical trauma has been shown to cause cochlear ossification, as demonstrated by histological studies [37, 38]. Cochlear dimensions and orientation are crucial for selecting patient-appropriate electrodes, as the depth of the array impacts both the preservation of residual hearing and the risk of causing trauma to it. Although the human cochlea reaches adult size before birth, it is known that the size of the cochlea varies between individuals and shows significant differences in shape, size and spiral properties of the cochlea [31, 39, 40]. Pietsch et al. [21] reported that the interindividual variability of the cochlea is based on the modiolus, which is shaped by neural structures that develop early in contrast to the scalar spaces.

The cochlear orientation may undergo postnatal changes depending on the changes in the skull base [17]. Erixon et al. [41] and Lloyd et al. [17] reported that the basal turn angle is highly variable from person to person. In different posterior tympanotomy modalities, mathematically calculated approaches for robot-assisted surgery provide optimal cochlear approach angles in a collision-free trajectory that provides easy access to the surgeon [15]. In the current study, the mean basal turn angle was 58.72°±8.49° consistent with the literature [17, 41] Lloyd et al. [17], reported that a wide basal turn angle can cause a difficult cochlear implantation in terms of electrode trauma and basilar membrane damage. The increased basal turn angle may make it difficult for the surgeon to detect the basal turn [17]. The current study found a high degree of significant positive correlation between the basal turn angle and the in-plane angle (*p* = 0.001, *r* = 0.859). It is thought that the wide basal turn angle causes a difficult cochlear implantation due to this correlation. In the present study, the mean value of the cochlea apical angle (CAA) was 33.04°±7.02°, and no significant correlation was found between CAA and electrode insertion depth degree. The angle between the line passing through the apex of the cochlea and the midsagittal plane is one of the angles that determine the orientation of the cochlea at the skull base. While deep insertion improves postoperative hearing performance, it may increase the risk of damage to the apical cochlear structures [41]. Therefore, preoperative evaluation of CAA in both robotic and conventional surgery is thought to be significant to preserve apical structures.

The facial recess is a surgically defined space rather than an anatomical space [42]. The traditional and most preferred approach for accessing the round window is the facial recess approach [43]. Until now, facial recess sizes have been studied by various methods [44–48] It is thought that it is essential to know these dimensions beforehand to avoid facial nerve and chordae tympani injuries. In the cadaveric study by Jain et al. [43] the mean FRA was found to be 26.91°±1.19°. In the CT based study performed by Jeon et al. [46] the mean FRA was found to be 18.40°±1.05°. In the current CT study, the mean FRA was 34.04°±5.88°. This angle was statistically significantly smaller in patient whose safe distance to the facial nerve could not be guaranteed with intraoperative imaging (*p* = 0.03). As the angle between the chorda tympani and the facial nerve increases, the size of the facial recess increases. Therefore, it is thought that a high FRA is very important for a safe trajectory, especially in robotic surgery. In our study, the average FRW was 3.01 ± 0.48 mm. In addition, the FRW was lower in *Subject 19* whose safe distance to the facial nerve could not be guaranteed with intraoperative imaging. The distance from the pre-calculated safe trajectory to the facial nerve should not be less than 0.4 mm as a criterion for suitability for robotic surgery [5]. However, no reference value has been reported for the facial recess sizes. We think that our present results are important in determining a reference FRS value for robotic surgery. In addition to these, the distance of the facial nerve (FN) to the mastoid surface, i.e., the depth of the FN within the temporal bone, is crucial in both robotic and conventional surgery. However, literature on findings related to the depth of the FN is quite limited. Aslan et al. [49] reported that in 90 HRCT images, the FN was 15 mm medial to the mastoid surface. Dai et al. [50] reported that the mean distance was 19.07 mm between the lateral edge of the mastoid process and the central point of the FN. They also mentioned that the FN is more laterally positioned in males compared to females [50]. Additionally, it is noted that the position of the FN in the temporal bone is influenced by mastoid pneumatization [49, 50]. In the current study, the mean DFNM was 17.60 ± 3.79 mm.

The round window anatomy of the cochlea is recently of interest for three major clinical applications and perhaps gene therapy may be a future one. The oldest clinical importance of the round window anatomy is associated to the intratympanic pharmacological treatments. In this sense it is a true therapeutic window for molecules under a molecular weight of 1000 MV can diffuse through the membrane and have a therapeutic effect for e.g. Meniere disease or sudden deafness. Of course, here is the second-round window membrane to account for in this implication. In a second use there are floating mass transducers who can use the RW for inverse sonance [51]. Some controversial therapies have been published on the plugging of the RW niche in the case of a third window phenomenon as given superior semicircular canal dehiscence [52]. The major clinical importance of the round window is of course in profound SNHL and the placement of cochlear implant arrays.

The round window, although its name indicates round, is an anatomical structure that can be quite variable in size and is often not round. The dimensions of the RW can be defined by height or, width or just, large and small diameter [53, 54]. In some patients, the distance defined as large diameter or height in some studies in the literature may be smaller than the small diameter or width. Therefore, it is thought that the nomenclature made so far is not anatomically correct. In order to avoid this confusion, in our study, the terms supero-inferior diameter and antero-posterior diameter were used for the first time for RW dimensions, taking into account the RW anatomical position. In the literature, the mean data range for RW supero-inferior diameter is 1.62–2.98 mm, while 1.15–1.66 mm for RW antero-posterior diameter is reported [25, 53–60]. In our 3D reconstruction study, antero-posterior diameter, and supero-inferior diameter of RW were found to be 1.60 ± 0.49 mm and 1.88 ± 0.37 mm, respectively. The differences in our averages with the literature may be due to demographic, gender and methodological differences. In addition, the supero-inferior diameter of RW of subject 17, which could not be fully inserted, was smaller than the other patients. This result suggests that the RW size may affect the full insertion of the electrode.

### Limitations

The number of patients in the current study is small, because of the pioneering character of this type of robotic surgery and cases are screened for suitable anatomy. Therefore, the group of converted robotic surgeries to conventional surgery is rather small making a comparative study of the anatomy difficult. A more thorough anatomical comparison should also include pediatric anatomy however the HEARO has no CE marking for pediatric cases under the age of 18.

## Conclusion

Hereby in our robotic surgery cases, we demonstrate that the dimensions of the facial recess and the orientation of the RW are the limiting factors of anatomical relationships. Therefore, preoperative evaluation of cochlear orientation is essential for safe cochlear access. In addition, a small round window may affect the full electrode insertion. The findings of this study would be a reference for future studies in achieving collision-free trajectory planning in robotic-assisted cochlear implant surgery.

## Data Availability

The data that support the results of this study are available on request from the corresponding author.
